# Smart grading of diabetic retinopathy: an intelligent recommendation-based fine-tuned EfficientNetB0 framework

**DOI:** 10.3389/frai.2024.1396160

**Published:** 2024-04-16

**Authors:** Vatsala Anand, Deepika Koundal, Wael Y. Alghamdi, Bayan M. Alsharbi

**Affiliations:** ^1^Chitkara University Institute of Engineering and Technology, Chitkara University, Rajpura, Punjab, India; ^2^School of Computer Science, University of Petroleum and Energy Studies, Dehradun, India; ^3^Ho Chi Minh City Open University, Ho Chi Minh City, Vietnam; ^4^Department of Computer Science, College of Computers and Information Technology, Taif University, Taif, Saudi Arabia; ^5^Department of Information Technology, College of Computers and Information Technology, Taif University, Taif, Saudi Arabia

**Keywords:** diagnosis, diabetic retinopathy (DR), health, healthy, pre-trained

## Abstract

Diabetic retinopathy is a condition that affects the retina and causes vision loss due to blood vessel destruction. The retina is the layer of the eye responsible for visual processing and nerve signaling. Diabetic retinopathy causes vision loss, floaters, and sometimes blindness; however, it often shows no warning signals in the early stages. Deep learning-based techniques have emerged as viable options for automated illness classification as large-scale medical imaging datasets have become more widely available. To adapt to medical image analysis tasks, transfer learning makes use of pre-trained models to extract high-level characteristics from natural images. In this research, an intelligent recommendation-based fine-tuned EfficientNetB0 model has been proposed for quick and precise assessment for the diagnosis of diabetic retinopathy from fundus images, which will help ophthalmologists in early diagnosis and detection. The proposed EfficientNetB0 model is compared with three transfer learning-based models, namely, ResNet152, VGG16, and DenseNet169. The experimental work is carried out using publicly available datasets from Kaggle consisting of 3,200 fundus images. Out of all the transfer learning models, the EfficientNetB0 model has outperformed with an accuracy of 0.91, followed by DenseNet169 with an accuracy of 0.90. In comparison to other approaches, the proposed intelligent recommendation-based fine-tuned EfficientNetB0 approach delivers state-of-the-art performance on the accuracy, recall, precision, and F1-score criteria. The system aims to assist ophthalmologists in early detection, potentially alleviating the burden on healthcare units.

## 1 Introduction

High blood glucose levels can lead to diabetic retinopathy, an eye disorder caused by damage to the retinal blood vessels. The retina is the layer of the eye responsible for visual processing and nerve signaling. Diabetic retinopathy often shows no early warning signs but can eventually cause blurred vision, floaters, and even blindness. Diabetic retinopathy can be either non-proliferative or proliferative, depending on the severity of the disease. Maintaining a healthy blood sugar level and scheduling routine eye exams can help stop or reduce the development of diabetic retinopathy. Early detection is key because treatment with laser therapy or surgery can prevent permanent loss of eyesight. Normal eye examinations are fundamental for individuals with diabetes to detect diabetic retinopathy and other eye complications (Nazir et al., 2019; Raja and Balaji, 2019). Diabetic retinopathy may be a potential consequence of diabetes, which can lead to permanent vision loss or visual deficiency if left untreated expeditiously. It is the leading cause of visual impairment in adults of working age in numerous economically developed nations. Individuals with diabetes, particularly those with uncontrolled blood sugar levels, are at a higher chance of developing diabetic retinopathy. The probability of an increase in complications of diabetes is observed for longer periods (Gayathri et al., 2021). Moreover, diabetic retinopathy may be a precursor to other diabetes-related issues such as nerve injury or kidney infection. Subsequently, it is fundamental for people with diabetes to oversee their blood sugar levels and cholesterol levels to maintain a strategic distance from this condition and other issues. Diabetic retinopathy can be viably treated on the probability that it is recognized and treated early.

Diabetic retinopathy is classified into two subtypes. In its earliest stage, diabetes can cause a disease known as non-proliferative diabetic retinopathy (NPDR). Exudates are deposits formed when injured blood vessels in the retina leak. In its earliest stages, NPDR rarely causes any noticeable symptoms or vision loss. Proliferative diabetic retinopathy (PDR) is a more severe form of the disease. In brief, PDR happens when new, fragile blood vessels form on the surface of the retina and have the potential to bleed into the vitreous, gel-like fluid that fills the inside of the eye. PDR can lead to total blindness if left untreated.

Early diagnosis of diabetic retinopathy is essential in avoiding permanent vision impairment or blindness. Untreated diabetic retinopathy, a condition that affects the retinal blood vessels, can cause severe vision loss or even blindness. DR often shows no signs in its early stages or only moderate signs such as impaired vision. However, if the disease worsens, it can cause significant vision loss, and by then, it may be too late for effective treatment. Diabetic retinopathy can be detected and treated early on if patients undergo routine eye examinations. Treatments such as laser therapy and injections can be used to halt or delay the growth of the disease if they are administered early on. In rare circumstances, surgery may be the only option.

By using deep learning techniques, automated systems for the early diagnosis of diabetic retinopathy have been created. Fundus photography is a typical technique used for capturing an image of the back of the eye. To train deep learning models, we may use large labeled datasets of fundus images that have been classified as either healthy or showing signs of diabetic retinopathy. After being trained, these algorithms can determine if a new fundus image is healthy or showing signs of diabetic retinopathy, and some can even assign a severity score to the condition. Deep learning has shown encouraging results in the early detection of diabetic retinopathy, and it has the potential to improve the efficacy and precision of screening programs, especially in areas with limited access to ophthalmologists (Li et al., 2019; Washburn, 2020).

Diabetic retinopathy can be diagnosed and managed with several different imaging techniques, including but not limited to the following:

In fluorescein angiography, a dye is injected into the arm, and the blood vessels in the retina are photographed as the dye travels through the body. It can be used to detect and monitor the growth of aberrant retinal blood vessels. Similarly, indocyanine green angiography, an invasive imaging modality, entails injecting a dye into the arm and then taking pictures of the retina while the dye travels through the blood arteries. Patients with diabetic retinopathy often undergo indocyanine green angiography to assess choroidal circulation. The high-frequency sound waves used in ultrasonography can be used to create images of the eye without causing any damage. It can detect and monitor changes in the retina, optic nerve, and other components of the eye.

The major contributions of this research are as follows:

- Four transfer learning models, namely, EfficientNetB0, ResNet152, VGG16, and DenseNet169, have been simulated for the two-class classification of fundus images for the diagnosis of diabetic retinopathy. For this diagnosis, two classes of fundus images having 3,200 images are considered.
- Furthermore, fine-tuning of pre-trained models has been performed by adding a global average pooling layer, a flatten layer, a dropout layer, and a dense layer to save time and resources and to improve the performance of the model.
- The performance of the best model has been compared with that of state-of-the-art techniques based on precision, recall, accuracy, and F1-score, in which EfficientNetB0 has outperformed other models.
- This research can help ophthalmologists gain further information, which will help them in the early detection and diagnosis of the disease.

The remainder of this article is structured as follows: Section 2 provides the literature review; Section 3 describes the proposed methodology; Section 4 shows the results of this study; and Section 5 provides the conclusion.

## 2 Literature review

Various researchers have used different techniques for diagnosing diabetic retinopathy from retinal images. Kaggle Asia Pacific Tele-Ophthalmology Society (APTOS), Messidor, Messidor-2, Kaggle eyePACS, STARE, HRF, Indian Diabetic Retinopathy Image Dataset (IDRiD), and DDR are only some of the datasets that have been used by researchers.

Anoop (2022) presented the convolutional neural networks (CNN) architecture for the binary class classification of DR and non-DR images. By comparison, using different hyperparameters, they obtained an accuracy (Acc) of 0.946, a sensitivity (Se) of 0.860, and a specificity (Sp) of 0.960. Pamadi et al. (2022) worked using the MobileNetV2 architecture for the binary and multimodal classification of DR images. They used the Kaggle APTOS dataset and attained an Acc of 0.780. Saranya et al. (2022) presented DenseNet models for the diagnosis of DR. They attained an Acc of 0.830 and a Pr of 0.99. Sanjana et al. (2021) worked using five different transfer learning models for the detection of DR with 1,115 fundus images consisting of two classes. They obtained an Acc of 0.861, a Se of 0.854, and a Sp of 0.875. Kumar and Karthikeyan (2021) used different models such as EfficientNet and Swin-Transformer with 3,600 fundus images and obtained an Acc of 0.864 on Swin-Transformer. Lahmar and Idri (2022) presented automatic two-class classification using 28 hybrid architectures. They combined four classifiers and seven deep learning algorithms and used Kaggle DR, Messidor-2, and APTOS datasets for the simulation, which produced the highest value of an Acc of 0.890 on the APTOS dataset.

El-Ateif and Idri (2022) used seven different pre-trained algorithms using APTOS and Messidor datasets. They obtained an Acc of 0.907 on the DenseNet121 architecture. Jiang et al. (2017) presented a CNN model architecture for the two-class classification of fundus images. They used 8,626 images for training and 1,925 images for validating the model and attained an Acc of 0.757. Rêgo et al. (2021) used the Inception-V3 model with 295 images. They obtained an Acc of 0.95%, followed by a Se of 0.808 and a Sp of 0.973. Kolla and Venugopal (2021) proposed a binary CNN that reduces the power and efficiency of retinopathy classification. They used the Kaggle dataset on DR images and obtained an Acc of 0.910. Kazakh-British et al. (2018) presented the training of an ANN using 400 fundus images. They obtained an increased Acc of 0.600. Elwin et al. (2022) used a deep learning algorithm for the diagnosis of the retina. They obtained Acc, Se, and Sp values of 0.9142, 0.9254, and 0.9142, respectively. Shorfuzzaman et al. (2021) used a weighted fusion of different models for the detection of DR by using three datasets, namely, APTOS, MESSIDOR, and IDRiD. They obtained a Pr of 0.970, a Se of 0.980, and an AUC of 0.978. Elswah et al. (2020) performed the diagnosis of DR using three steps. In the first step, fundus images were pre-processed using augmentation and normalization. In the second step, the ResNet-based CNN model was used for the diagnosis, followed by the classification of DR fundus images. They obtained an Acc of 0.866.

Sakaguchi et al. (2019) presented the graph neural network for the diagnosis of DR. They used three datasets and obtained accuracy values of 0.783, 0.774, and 0.759. Shaik and Cherukuri (2022) presented the Hinge Attention Network for DR diagnosis. They used the transfer learning-based VGG16 network architecture. The authors worked using Kaggle APTOS 2019 and ISBI IDRiD datasets and obtained the accuracy values of 85.54 and 66.41%, respectively. AbdelMaksoud et al. (2022) combined the EyeNet and DenseNet models for the diagnosis of DR and used four datasets, namely, EyePACS, Indian diabetic retinopathy image dataset, MESSIDOR, and Asia Pacific Tele-Ophthalmology Society (APTOS 2019), and resized the images to 256 ^*^ 256. The proposed model achieved an Acc of 0.912, a Se of 0.96, and a Sp of 0.69. Thota and Reddy (2020) used the VGG16 pre-trained neural network for the diagnosis of DR and achieved the values of Acc, Se, and Sp of 0.740, 0.80, and 0.65, respectively. Barhate et al. (2020) worked using three pre-trained models, namely, VGG19, VGG16, and AlexNet. They proposed the VGG autoencoder network and worked using the EyePACS dataset. They obtained an Acc of 0.762 with the proposed model.

Kwasigroch et al. (2018) proposed deep CNN for the diagnosis of DR. They achieved an Acc of 82% and a Kappa score of 0.776. Wang et al. (2018) presented different pre-trained networks for the diagnosis of DR. They used 166 fundus images obtained from the Kaggle dataset and obtained Acc values of 37.43% on AlexNet, 50.03% on VGG16, and 63.23% on InceptionNetV3. Zhou et al. (2018) proposed a multi-cell architecture that boosts the training time and increases the classification Acc to 0.632. Shrivastava and Joshi (2018) presented a CNN-based network for the detection of DR in fundus images and used the InceptionV3 CNN architecture, and the features were extracted using the Support Vector Machine (SVM). They obtained an Acc of 0.877 on binary class and 0.818 on multi-class classifications. Maistry et al. (2020) proposed the CNN for the diagnosis of DR from fundus images. They carried out the analysis on the EyePACS dataset and obtained an Acc of 0.869 and an F1-score of 0.80. Khaled et al. (2020) presented the cascade model to detect DR and classify it into four stages. They used 61,248 retinal fundus images. They had obtained a Sp of 96.1%. Qian et al. (2021) combined the Res2Net and DenseNet models for the diagnosis of DR. They achieved the Acc and Kappa scores of 0.832 and 0.8, respectively. Xiao et al. (2021) presented an Inception module for DR diagnosis. They obtained an Acc of 88.24% and a Se of 99.43%. In this study, an intelligent fine-tuned EfficientNetB0 model has been proposed for the diagnosis of DR.

## 3 Proposed methodology

**Figure 1:**
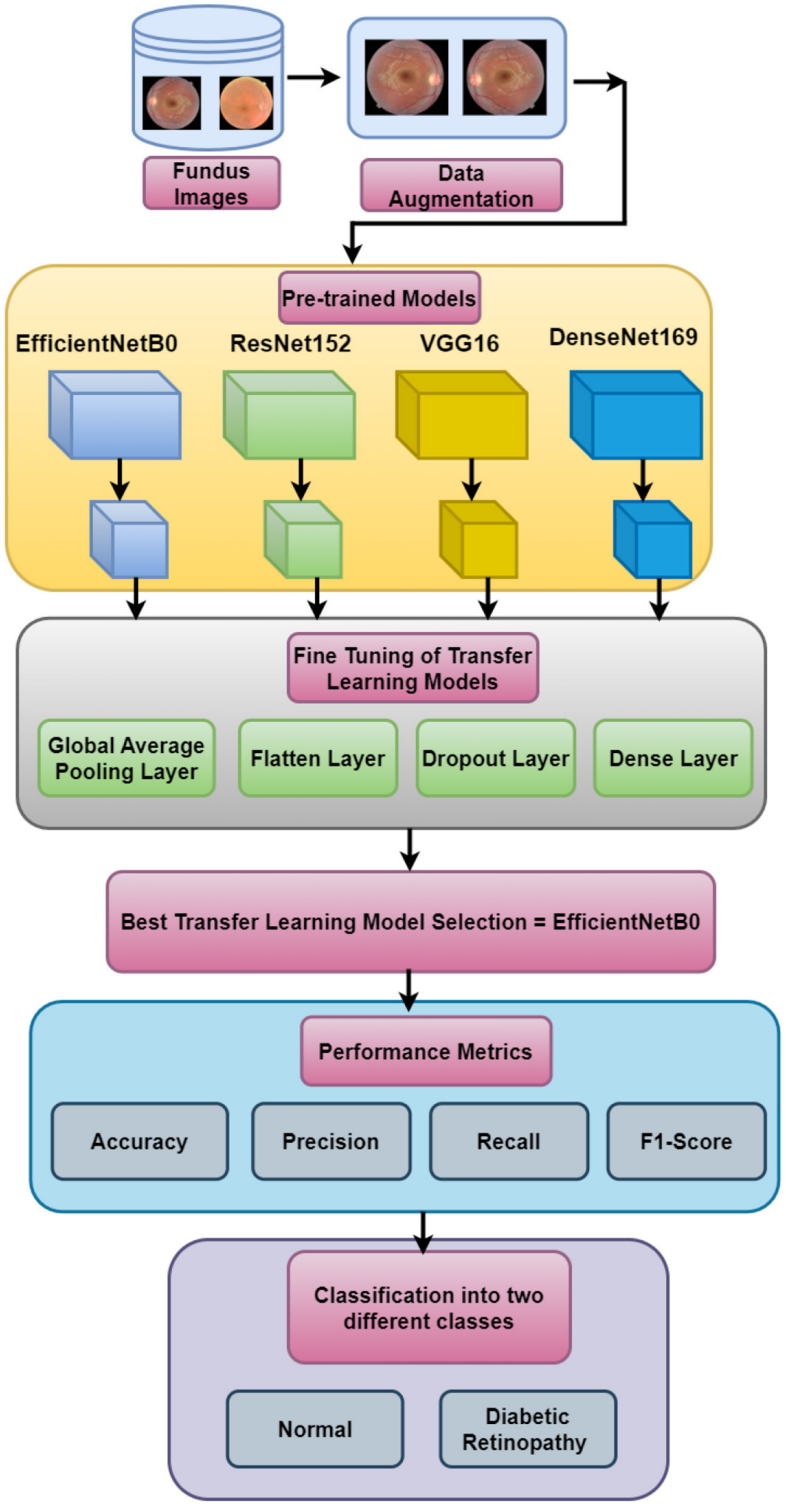
Proposed methodology.

This research presents the simulation of four transfer learning models, namely, EfficientNetB0, ResNet152, VGG16, and DenseNet169, for the two-class classification of fundus images to detect diabetic retinopathy. Figure 1 depicts the input dataset that includes fundus images. The data augmentation techniques are applied to the original dataset. The next block represents the simulation using four pre-trained models. Furthermore, fine-tuning of the pre-trained models has been performed by adding the global average pooling layer, the flatten layer, the dropout layer, and the dense layer to save time and resources and improving the performance of the model. Out of the four pre-trained models, EfficientNetB0 is selected as the best-performing model. The performance of the best model has been compared with that of state-of-the-art techniques based on precision, recall, accuracy, and F1-score, in which EfficientNetB0 has outperformed.

### 3.1 Input dataset

**Figure 2:**
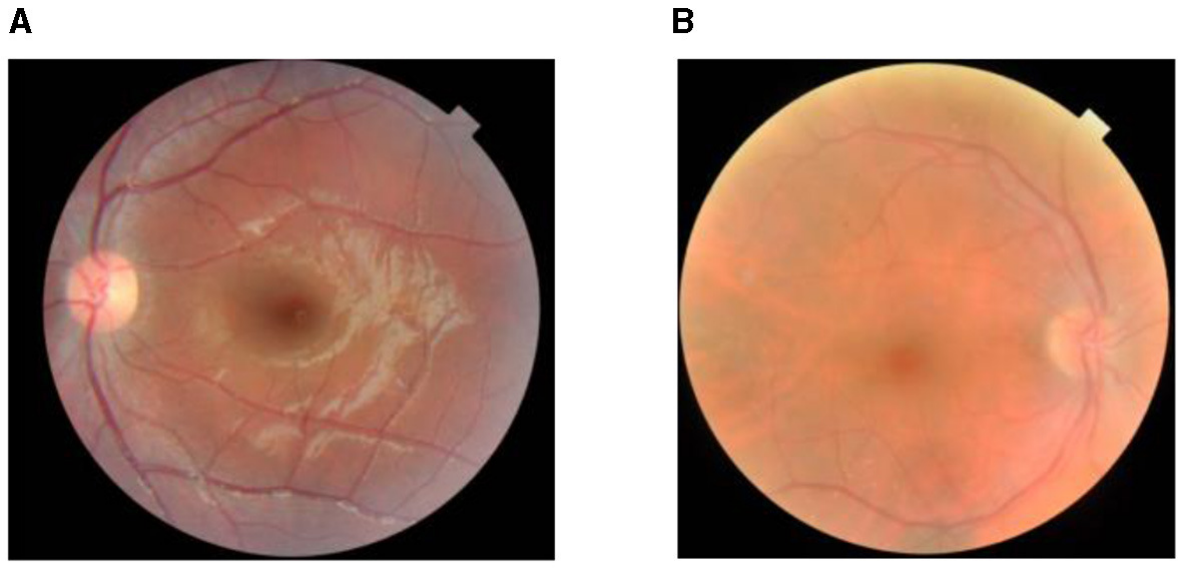
Dataset sample: **(A)** cataract and **(B)** non-cataract.

The proposed work has been validated using the retinal disease dataset that is obtained from public sources (Pachade et al., 2021). The dataset consists of 3,200 fundus images that are captured using three different fundus cameras with the help of two senior retinal experts. Out of the 3,200 images, 1,920 images are used for training, 640 images are used for validation, and 640 images are used for testing. In total, 60% of the data are used for training, 20% for testing, and the other 20% for validation purposes. The sample images are shown in Figure 2. The study uses open data, which are data that are freely available to the public. Since the data used in this study are accessible to the public and do not involve any private or sensitive information, they fall under the category of research that is exempt from the oversight of the Institutional Review Board (IRB). Therefore, no IRB approval was required for this study. However, all analyses were conducted in accordance with ethical standards and guidelines for research integrity.

### 3.2 Data augmentation

**Figure 3:**
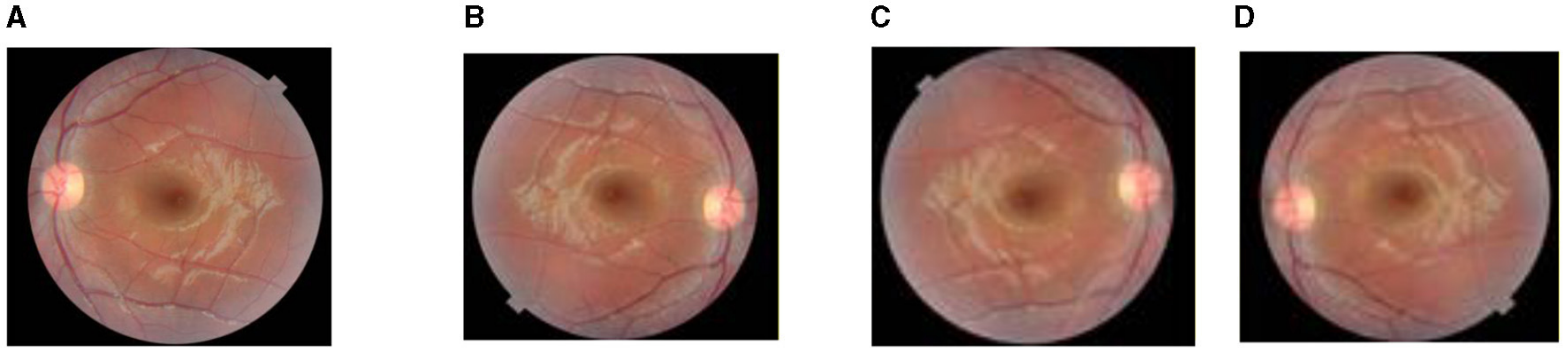
Images after augmentation: **(A)** original image, **(B)** rotation (180°), **(C)** horizontal flipping, and **(D)** vertical flipping.

Figure 3 shows the data augmentation techniques that are applied to the original dataset to obtain a different variety of images. Figure 3A shows the original fundus image, Figure 3B shows the 180-degree rotated image, Figure 3C shows the horizontal flipping, and Figure 3D shows vertical flipping.

### 3.3 Prediction using pre-trained models

Common deep-learning architectures used for computer vision tasks include EfficientNetB0, ResNet152, VGG16, and DenseNet169. Each of these algorithms has its special traits and advantages when it comes to identifying and categorizing images.

EfficientNetB0 has an outstanding record in the use of computational resources. Its precision is remarkable given that the model is so small and easy to run. The architecture scales in depth, width, and resolution to maximize performance under varying conditions of availability.

ResNet152, in contrast to EfficientNetB0, is a considerably more in-depth and complicated model. Because of its basis in the residual learning framework, even extremely deep neural networks can be trained with relative ease. With its 152 layers, ResNet152 excels at image classification. However, it has higher computing requirements because of its increased depth.

The VGG16 architecture for CNNs is another popular choice. It has 16 layers of movable weights and is renowned for its uniformity and ease of use. VGG16 has performed exceptionally well in several computer vision competitions, notably the ImageNet challenge. Its simple design makes it easy to grasp and put into practice.

DenseNet169 is a convolutional neural network (CNN) architecture with a substantial focus on feature reuse and a preference for steep gradient descents. It implements skip links between layers, providing instant access to feature maps in higher-level ones. This dense connectivity structure decreases the likelihood of disappearing gradients and increases accuracy by facilitating the efficient flow of information and gradient propagation across the network (Trivedi et al., 2021; Ramesh et al., 2022).

DenseNet169 uses a dense connection to promote feature propagation, whereas VGG16 is well-known for its simplicity and solid performance. EfficientNetB0 provides an excellent compromise between efficiency and accuracy. The available computational resources, the quantity of the dataset, and the desired accuracy all play a role in deciding the model to be used. Table 1 shows the parameters of pre-trained models.

**Table 1:** Pre-trained models.

**Model**	**Layers**	**Parameters (in millions)**	**Input layer size**	**Output layer size**
EfficientNetB0	10	8.4	224 x 224 x 3	(2, 1)
ResNet152	164	60.4		
VGG16	16	138		
DenseNet169	169	27		

## 4 Results and discussion

This section includes all the findings from various modeling studies. Pre-trained architectures with varying numbers of epochs and confusion matrix parameters are compared experimentally (Sharma and Koundal, 2018; Bhattacharya et al., 2022; Gupta et al., 2023; Koundal et al., 2023).

### 4.1 Epoch-wise assessment of different models

Table 2 shows the epoch-wise assessment of four models. The model with the lowest loss across all epochs is VGG16, with a loss value of 0.0080 at epoch 12. The model with the highest loss is DenseNet169, with a loss of 0.4358 at epoch 3. VGG16 achieves the highest accuracy among all models, reaching 0.9974 at epoch 12. DenseNet169 has the lowest accuracy of 0.9161 at epoch 3. VGG16 achieves the highest AUC value among the models, with a peak of 0.9999 at epoch 12. EfficientNetB0 and ResNet152 also show excellent AUC values, with EfficientNetB0 reaching 0.9949 and ResNet152 reaching 0.9912. The model with the highest precision is VGG16, which achieves a precision of 0.9920 at epoch 12. The lowest precision among the models is achieved by DenseNet169, with a precision of 0.7929 at epoch 3. VGG16 achieves the highest recall among the models, reaching 0.9947 at epoch 12. DenseNet169 has the lowest recall, with a value of 0.7739 at epoch 3. In summary, VGG16 consistently performs well across all the metrics, showing the lowest loss, the highest accuracy, AUC, precision, and recall. EfficientNetB0 and ResNet152 also exhibit strong performance, particularly in terms of the AUC. DenseNet169 generally has lower performance than the other models in terms of accuracy, precision, and recall.

**Table 2:** Epoch-wise assessment of four models.

**Model**	**Epochs**	**Loss**	**Accuracy**	**AUC**	**Precision**	**Recall**
EfficientNetB0	5	0.2696	0.9417	0.9503	0.8455	0.8590
	10	0.1498	0.9625	0.9797	0.8979	0.9122
	15	0.0536	**0.9854**	0.9949	0.9703	0.9548
ResNet152	3	0.4042	0.9490	0.9465	0.8639	0.8777
	6	0.0723	0.9859	0.9896	0.9704	0.9574
	9	0.0547	**0.9865**	0.9912	0.9679	0.9628
VGG16	4	0.2698	0.9531	0.9583	0.8803	0.8803
	8	0.0234	0.9911	0.9980	0.9761	0.9787
	12	0.0080	**0.9974**	0.9999	0.9920	0.9947
DenseNet169	3	0.4358	0.9161	0.9143	0.7929	0.7739
	6	0.0815	**0.9729**	0.9904	0.9355	0.9255
	8	0.1238	0.9625	0.9815	0.9086	0.8989

Bold values are the best values of accuracy on different models.

EfficientNetB0 demonstrates consistent improvement in performance with an increase in the number of epochs. It achieves high accuracy, AUC, precision, and recall values, indicating an overall good performance. Among the evaluated metrics, EfficientNetB0 achieves the highest precision and recall at epoch 15. EfficientNetB0 shows competitive performance compared to the other models in the table. ResNet152 also shows improvement in performance with an increase in the number of epochs. It achieves high accuracy, AUC, precision, and recall values. ResNet152 performs particularly well in terms of AUC, with high values across epochs. The model exhibits competitive performance overall, similar to EfficientNetB0. VGG16 consistently demonstrates strong performance across all evaluated metrics. It achieves the lowest loss, the highest accuracy, AUC, precision, and recall values among all models. VGG16 exhibits consistent improvement with an increase in the number of epochs. The model consistently outperforms the other models given in the table. DenseNet169 shows relatively stable performance across epochs, with some fluctuations in loss and other metrics. While it achieves lower values compared to the other models in terms of accuracy, precision, and recall, it still demonstrates competitive performance. DenseNet169 performs relatively well in terms of the AUC, although not as high as VGG16 and ResNet152.

### 4.2 Binary class-wise assessment of confusion matrix parameters

Table 3 shows the class-wise assessment in terms of confusion matrix parameters. For the non-infected (N-IN) disease class, EfficientNetB0 achieves impressive results with a precision of 0.93, a recall of 0.94, an F1-score of 0.94, and an accuracy of 0.91. These metrics indicate that EfficientNetB0 performs exceptionally well in correctly identifying and classifying instances of the N-IN disease class. The high precision and recall values demonstrate a strong ability to accurately predict positive cases while minimizing false positives and false negatives. The F1-score, which combines precision and recall, further reflects the overall effectiveness of the model. ResNet152 also performs well for the N-IN disease class with a precision of 0.93, a recall of 0.91, an F1-score of 0.92, and an accuracy of 0.88. Although slightly lower than that of EfficientNetB0, these scores still indicate a robust performance in disease classification. ResNet152 shows good potential in accurately identifying instances of the N-IN disease class, and the high accuracy value suggests a reliable overall prediction ability. VGG16, on the other hand, achieves slightly lower scores than those in the previous models, with a precision of 0.92, a recall of 0.93, an F1-score of 0.92, and an accuracy of 0.89 for the N-IN disease class. While these metrics demonstrate a reasonably accurate classification, they indicate a slightly lower performance than EfficientNetB0 and ResNet152. However, VGG16 still shows promise in disease classification tasks. DenseNet169 showcases strong performance for the N-IN disease class, with a precision of 0.94, a recall of 0.93, an F1-score of 0.94, and an accuracy of 0.90. Similar to EfficientNetB0, DenseNet169 achieves high precision, recall, and F1-score values, indicating a robust ability to correctly classify instances of the N-IN disease class. The accuracy score also suggests reliable overall predictions. In conclusion, based on the available information, EfficientNetB0 and DenseNet169 emerge as the top performers for the N-IN disease class, exhibiting high precision, recall, F1-score, and accuracy values. ResNet152 follows closely behind with slightly lower scores, while VGG16 shows comparatively lower but still promising results. These findings highlight the strengths of EfficientNetB0 and DenseNet169 in disease classification tasks, emphasizing their potential for accurate identification and classification of instances belonging to the N-IN disease class.

**Table 3:** Binary class-wise assessment of confusion matrix parameters.

**Models**	**Disease Class**	**Precision**	**Recall**	**F1-score**	**Accuracy**
EfficientNetB0	N-IN	0.93	0.94	0.94	0.91
	IN	0.76	0.73	0.75	
ResNet152	N-IN	0.93	0.91	0.92	0.88
	IN	0.69	0.73	0.71	
VGG16	N-IN	0.92	0.93	0.92	0.89
	IN	0.71	0.68	0.70	
DenseNet169	N-IN	0.94	0.93	0.94	0.90
	IN	0.75	0.78	0.76	

### 4.3 Comparison curves

**Figure 4:**
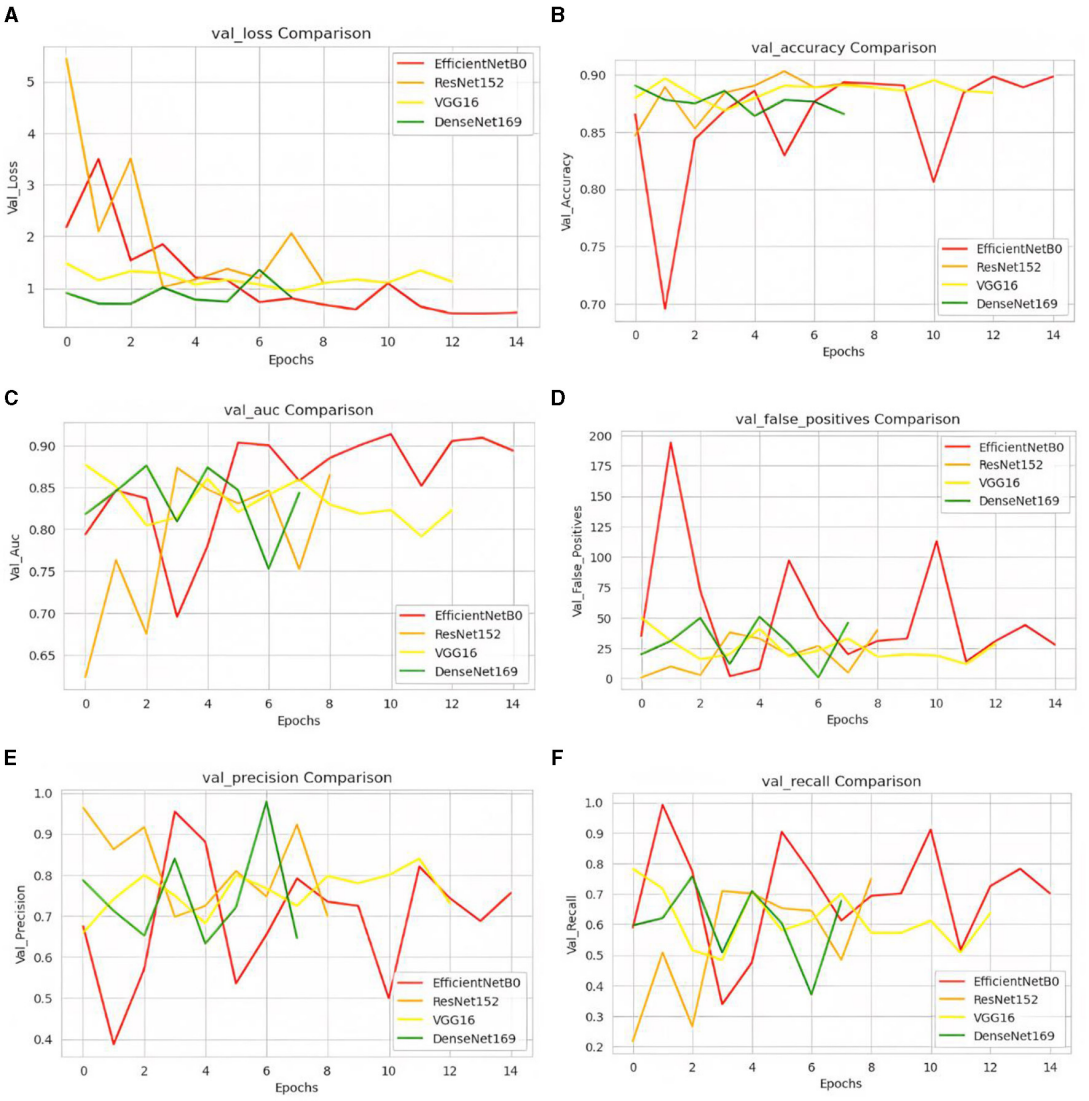
A comparison of transfer learning models: **(A)** loss, **(B)** accuracy, **(C)** AUC, **(D)** false positives, **(E)** precision, and **(F)** recall.

After all the evaluations, different comparison curves are obtained. Figure 4A shows the validation loss comparison curve for the four pre-trained models. It can be observed that the value of the loss is 0.5 in the case of the EfficientNetB0 model. Figure 4B shows the values of accuracy for all four pre-trained models. For the EfficientNetB0 model, the value of accuracy is 0.91, followed by 0.90 for DenseNet169. The value of accuracy is 0.89 for VGG16 and 0.88 for ResNet152. Figure 4C shows the AUC comparison for all four pre-trained models. The values of AUC are 0.90, 0.86, 0.82, and 0.84 for EfficientNetB0, ResNet152, VGG16, and DenseNet169, respectively. Figure 4D shows the comparison of false positives for all four pre-trained models. Figure 4E shows the comparison of the precision with the value of EfficientNetB0 between 0.7 and 0.8. Figure 4F shows the values of recall for four pre-trained models. The EfficientNetB0 model shows a recall of 0.70.

From Figure 4, it can be concluded that EfficientNetB0 has outperformed all the other models in terms of accuracy. The value of the highest accuracy obtained is 0.91.

### 4.4 A comparison with the state-of-the-art methods

The researchers used different transfer learning models for the diagnosis of DR as shown in Table 4. The different transfer learning techniques were used by researchers to achieve different values of performance metrics. The proposed fine-tuned EfficientNetB0 model has outperformed the other models and obtained an accuracy of 0.91.

**Table 4:** A comparison with state-of-the-art methods.

**References**	**Technique**	**Dataset/number of images**	**Performance parameters**
Pamadi et al. (2022)	MobileNetV2	Kaggle Aptos/-	Acc = 0.780
Saranya et al. (2022)	DenseNet	-	Acc = 0.830, Pr = 0.99
Sanjana et al. (2021)	Transfer learning models	Fundus/1,115	Acc = 0.861, Se = 0.854, Sp = 0.875
Kumar and Karthikeyan (2021)	Swin-Transformer	Fundus/3,600	Acc = 0.864
Lahmar and Idri (2022)	Combination of classifiers and deep learning algorithms	Kaggle DR, Messidor-2, and APTOS	Acc = 0.890
El-Ateif and Idri (2022)	Transfer learning models	APTOS and Messidor datasets	Acc = 0.907
Jiang et al. (2017)	CNN	8,626 images and 1,925 images for validating	Acc = 0.757
Rêgo et al. (2021)	Inception-V3	295	Acc = 0.95, Se = 0.808, Sp = 0.973
Kolla and Venugopal (2021)	CNN	-	Acc = 0.910
Kazakh-British et al. (2018)	ANN	400	Acc = 0.600
Proposed model	EfficientNetB0	Fundus/3200	Acc = 0.91

## 5 Conclusion and future work

In summary, identifying diabetic retinopathy in diabetic patients in fundus images is an essential step in its early detection and treatment, as it may potentially lead to blindness. Diabetic retinopathy diagnosis has been greatly aided by the development of image processing techniques and the proliferation of high-quality fundus images. Many different CAD systems have emerged to aid ophthalmologists in the analysis of fundus images over the years. Automated detection and grading of diabetic retinopathy lesions is achieved by the use of a wide range of image analysis techniques in these systems.

Diabetic retinopathy diagnosis has come a long way, but there are still limitations and room for development. First, validating and fine-tuning these algorithms on large-scale datasets is crucial because the performance of existing systems can vary between datasets. Second, various retinal illnesses may coexist, making it necessary to consider both validation and fine-tuning when interpreting fundus images for a diagnosis of diabetic retinopathy. The proposed intelligent recommendation-based fine-tuned EfficientNetB0 model has also been compared with state-of-the-art models in terms of accuracy. From the results, it is analyzed that the proposed model has outperformed the other transfer learning models and state-of-the-art models in terms of accuracy. The proposed intelligent recommendation-based fine-tuned EfficientNetB0 model will alleviate the economic burden on healthcare units. The proposed framework may aid medical practitioners and ophthalmologists in the detection and tracking of retinal illnesses, which in turn may lead to more timely treatment and better patient outcomes. Because of the flexibility of the transfer learning method, it might be incorporated into pre-existing telemedicine systems for use in remote screening and diagnosis.

The research findings on diabetic retinopathy have significant practical implications for medical diagnostics and patient care. By using deep learning models, such as CapsNet-Random Walrus (CapsNet-RW), for the automated detection and classification of diabetic retinopathy from retinal images, healthcare providers can enhance the efficiency and accuracy of diagnosis. This can lead to early detection and intervention, which are crucial in preventing vision loss and improving patient outcomes. Additionally, the use of such models can help in reducing the burden on healthcare systems by optimizing resource allocation and improving access to timely care for diabetic patients.

## Data availability statement

The original contributions presented in the study are included in the article/supplementary material, further inquiries can be directed to the corresponding author.

## Author contributions

VA: Conceptualization, Data curation, Methodology, Software, Writing – original draft, Writing – review & editing. DK: Formal analysis, Investigation, Visualization, Writing – review & editing. WA: Conceptualization, Formal analysis, Methodology, Writing – review & editing. BA: Conceptualization, Resources, Supervision, Writing – review & editing.
